# Integrative Modeling of Macromolecular Assemblies from Low to Near-Atomic Resolution

**DOI:** 10.1016/j.csbj.2015.08.005

**Published:** 2015-09-03

**Authors:** Xiaojun Xu, Chunli Yan, Robert Wohlhueter, Ivaylo Ivanov

**Affiliations:** Department of Chemistry, Center for Diagnostics and Therapeutics, Georgia State University, Atlanta, GA 30302, USA

**Keywords:** Integrative modeling, Hybrid modeling, Molecular dynamics flexible fitting (MDFF), Small angle X-ray scattering (SAXS), Electron microscopy (EM), DNA replication and repair

## Abstract

While conventional high-resolution techniques in structural biology are challenged by the size and flexibility of many biological assemblies, recent advances in low-resolution techniques such as cryo-electron microscopy (cryo-EM) and small angle X-ray scattering (SAXS) have opened up new avenues to define the structures of such assemblies. By systematically combining various sources of structural, biochemical and biophysical information, integrative modeling approaches aim to provide a unified structural description of such assemblies, starting from high-resolution structures of the individual components and integrating all available information from low-resolution experimental methods. In this review, we describe integrative modeling approaches, which use complementary data from either cryo-EM or SAXS. Specifically, we focus on the popular molecular dynamics flexible fitting (MDFF) method, which has been widely used for flexible fitting into cryo-EM maps. Second, we describe hybrid molecular dynamics, Rosetta Monte-Carlo and minimum ensemble search (MES) methods that can be used to incorporate SAXS into pseudoatomic structural models. We present concise descriptions of the two methods and their most popular alternatives, along with select illustrative applications to protein/nucleic acid assemblies involved in DNA replication and repair.

## 1 Introduction

The structures of complex biological assemblies command considerable attention, since critical cellular activities are more often than not carried out by such assemblies rather than by a single macromolecular component. A high-resolution structural model of an assembly is often crucial to understanding its function; and biological mechanisms can be deduced from a detailed view of the structure and interactions of components in an assembly. Structures at atomic resolution are usually obtained through X-ray crystallography or nuclear magnetic resonance (NMR) spectroscopy. However, the size and flexibility of macromolecular assemblies often pose technical difficulties, confounding structural elucidation and impeding mechanistic exploration by conventional methods. Cryo-electron microscopy (cryo-EM) is one of the most promising techniques for elucidating larger macromolecular complexes but until recently it was only capable of generating structural models at resolutions of 8–20 Å [1] – substantially lower than routine X-ray crystallography. Better resolution (3.5–4.5 Å) was reported only for complexes with high symmetry and stability [2–4]. Not until very recently have the advances in high-resolution image-capturing hardware [5] and image-processing technology [6] enabled cryo-EM to yield near-atomic resolution maps [7,8]. With the new technologies, the structure of a mammalian TRP channel, TRPV1, was successfully determined at a resolution of 3.4 Å, for the first time reaching side-chain resolution for a membrane protein without crystallization [9,10]. In 2014, the success of cryo-EM was boosted by many other explorations, resulting in 3.0–5.0 Å resolution structures of β-galactosidase [11], membrane proteins [12–14] and ribosomal machineries [15] and leading to the notion of “resolution revolution” in single particle cryo-EM [16]. Recently, Campbell et al. reported a cryo-EM reconstruction of 2.8 Å for the 700 kDa *Thermoplasma acidophilum* 20S proteasome [17]. Furthermore, in 2015 the Subramaniam group at the National Cancer Institute further refined a β-galactosidase EM structure to an unprecedented 2.2 Å resolution [18], whereby the authors were able to identify densities of structural water molecules and ions, and demonstrated it is rather the intrinsic flexibility of the target molecule/complex and the quality of the specimen than the image-capturing or processing technologies that prevented achieving resolution close to 2 Å by cryo-EM. Apart from the breakthrough of near-atomic resolution, cryo-EM offers significant advantages in not requiring the high concentration of protein/complex that X-ray crystallography demands [19]. Nor does it require preparation of macroscopic crystals, since individual complexes are preserved in a frozen hydrated state on an EM grid. Thus, cryo-EM visualizes a structure more akin to that “in solution”, and probably of more relevance to in vivo conditions [19]. Given all of these exciting developments, cryo-EM stands poised to overtake X-ray crystallography and play an even more prominent role in the visualization of macromolecular complexes.

Other technologies also generate spatial envelopes of biological molecules or assemblies e.g. negative stain electron microscopy (EM) and small angle X-ray scattering (SAXS), while detailed interaction profiles are accessible through methodologies like chemical footprinting, cross-linking, fluorescence resonance energy transfer (FRET), mass spectrometry (MS), proteomics studies, and so on [20,21]. Though both shape and interactions often contribute to modeling a complex, the results from these methods are largely heterogeneous and dispersed in the literature. Therefore, an integrative modeling approach capable of combining these heterogeneous data and translating them into a uniform structural representation would be valuable in advancing our understanding of the relevant biological functions of these assemblies. Incorporating information from such diverse approaches may in fact lead to a highly useful model in less time and effort than by the conventional means of X-ray crystallography or NMR spectroscopy. And this may be the only means of arriving at a useful model. Moreover, the resulting model may be more useful to experimentalists, in that, by consolidating diverse experimental data, it may generate new hypotheses directly amenable to experimental tests. A notable example of the power and utility of integrative modeling methods was given by an elegant study by Alber et al., which elucidated the architecture of the nuclear pore complex (NPC) using a combination of diverse high-quality proteomic and structural data [22]. The advance was made possible by an integrative modeling platform IMP. IMP provides software tools to represent almost any conceivable combination of experimental data (e.g. relative positions of protein domains, mutational data on residue contacts, shape information from SAXS envelopes, EM densities and symmetry information). This data could even be of a type not normally used for structure determination or ambiguous in terms of structural interpretation. This diverse data is subsequently converted to spatial restraints, which collectively determine a scoring function. A structural ensemble is then generated and analyzed, which optimally satisfies the scoring function. The considerable freedom to mix and match modules in IMP allows the seamless construction of new hybrid modeling protocols. The major advantage of IMP lies in the flexible nature of the code, written as a software framework – a collection of independent modules in C++ and Python. IMP also provides interfaces for developers to introduce new scoring functions, sampling schemes, analysis methods, model representations and integrative modeling applications [23].

To start integrative modeling, all relevant data from different lines of experimental, physical, bioinformatics, and statistical studies have to be pooled together for close examination. Upon a proper choice of the resolution with which the system of interest will be defined in the model, the applicable data that were collected in the first stage would have to be translated into spatial restraints on part or all of the system. For example, a residue–residue contact can be incorporated by applying a harmonic constraint on the distance between these two residues, and a cryo-EM density map can be used to generate a 3D-grid based function to bias the system being modeled to evolve toward it. To sample these constrained functions all together, various methods can then be applied, such as molecular dynamics (MD), Monte Carlo (MC), Brownian dynamics, and docking. In the end, an ensemble of models is generated for analysis and refinement toward a final model. Recent successes in implementing integrative modeling include a variety of systems, utilizing experimental data from X-ray, NMR, cryo-EM and SAXS [20]. These successes have contributed many innovative insights into biomolecular assemblies, and generated much interest in the approach. Karca et al. have comprehensively reviewed how different types of experimental data can be translated into restraints, suggesting four categories of restraints e.g. binding sites, distance, orientation, and shape, operating at a high level of abstraction [21]. When no high-resolution experimental structure (or structures from closely homologous organisms) are available, cryo-EM maps can still be used for secondary structure element identification using computational tools such as SSHunter [24], ab initio protein modeling using EM-fold [25], de novo protein structure prediction using RosettaCM [26,27]. In this review we concentrate on cryo-EM- and SAXS-based integrative modeling using atomistic MD simulation.

DNA replication and repair are fundamentally important biological processes and involve multiple protein-DNA complexes. The detailed structures of many of these complexes, however, are difficult to obtain through X-ray or NMR studies, due to their large size and intrinsic flexibility. Meanwhile, a great number of related experimental results, including X-ray crystal structures, biochemistry and biophysical signatures of various components, are accessible. This extensive body of information provides a favorable scenario in which to apply the integrative modeling approach. The modeling of the human Rad9–Hus1–Rad1/FEN1/DNA ternary complex [28] is reviewed here to illustrate the MDFF method [29] utilizing a negative stain EM density map. Other applications, in which the conformational space of ubiquitinated and/or SUMOylated Proliferating Cell Nuclear Antigen (PCNA) is explored, are also presented as a guide to incorporating experimental SAXS data into a hybrid modeling protocol [30,31].

## 2 Methods

### 2.1 Molecular Dynamics Flexible Fitting

Although the resolution of current cryo-EM methodology is generally not comparable to that of X-ray crystallography [1], cryo-EM is routinely capable of providing coarse structural information on macromolecular complexes, and in a biologically more realistic environment, perhaps even capturing different functional states [32]. Combining atomistic detail from crystal structures with a cryo-EM density map provides complementarity and enhances the model construct that might be deduced from each set of data alone. Methods developed for fitting atomic structures into cryo-EM maps can be divided generally into rigid-body docking and flexible fitting. Rigid-body docking (also often called rigid-body fitting), refers to the process of placing the atomic structure entity in the corresponding part of the cryo-EM density map as a rigid-body. Automated rigid-body docking approaches maximize the cross-correlation between the experimental cryo-EM density map and a simulated density map of the protein complex by performing an exhaustive search over a six-dimensional parameter space (three translational and three rotational degrees of freedom of the system) [33,34]. Fast Fourier Transform (FFT) is introduced to reduce computational complexity by transforming the translational degrees of freedom into Fourier space, leaving only the three rotational degrees of freedom to be evaluated in real space [35]. Other improvements including local cross-correlation (LCC) score [36], core-weighted (CW) cross-correlation score [37], vector quantization [38], and geometric hashing [39] were introduced subsequently to rigid-body docking to either customize the docking process or further improve computational efficiency. Flexible fitting has an advantage in that reasonable conformational variations are allowed in the fitting process, so as to give better correlation between the cryo-EM map and the modeled structure. A variety of flexible fitting methods have been developed in recent years, based on different mathematical flavors, including real-space refinement upon segmented rigid-body docking [40,41], normal-mode calculation based on optimization of the correlation between structure and map [42], vector quantization based coarse-grained model fitting [38], etc. More recently, a Monte Carlo search and simulated annealing molecular dynamics-based fitting method has been developed [43]. Other methods have applied external forces proportional to the gradient of the EM-map (implemented in MDFF) [44] or the gradient of the cross-correlation coefficient between the structure and the EM-map [45,46] along with MD simulations to guide the atoms into high-density regions of an EM-map. Among these different approaches, the MDFF method has gained popularity due to its simple implementation and its seamless compatibility with MD simulations.

The MDFF method was developed on top of classical MD simulation, in which a potential energy function, also known as the MD force field (*U*_MD_), is used to describe the interactions between atoms. Upon computing from Umd the forces experienced by the atoms, MD iteratively solves the Newtonian equations of motion and provides atomistic details of motions in the system. In MDFF, *U*_MD_ preserves all the physical parameters, thereby preventing the resulting structure from straying into a non-physical state. Two extra terms are added to the classical MD potential energy function in MDFF, *U*_EM_ and *U*_ss_.

*U*_EM_ is converted from the EM map and used to bias the atoms into the corresponding EM density region:

(1) $$U_{EM}\left(R\right)=\sum_{j}w_{j}V_{EM}\left(r_{j}\right)$$

where *w*_*j*_ is the weighting factor (usually set to the atomic mass) for atom *j* of coordinate *r*_*j*_. *V_EM_* is defined as the following:

(2) $$V_{EM}\left(r\right)=\left\{\begin{array}{cc} \xi & \left[1-\frac{\phi \left(r\right)-\phi_{thr}}{\phi_{\max}-\phi_{thr}}\right]\\ \xi & \end{array}\begin{array}{c} if\phi \;\left(r\right)\;\geq \phi_{thr}\\ if\phi \;\left(r\right)\;<\phi_{thr} \end{array}\right)$$

*ϕ*(*r*) is the potential converted from the cryo-EM density. *ϕ*_*max*_ is the maximum value in the given density map. *ϕ*_*thr*_ is the threshold value chosen to reduce the solvent influence. *ξ* is the scaling factor, applied uniformly to the biasing potential generated from the cryo-EM map. By varying the scaling factor the relative weight of the EM density biasing potential can be increased to ensure closer conformance of the model to the map. Conversely, *ξ* can be decreased in cases where it is necessary to prevent over-fitting.

*U*_ss_ is a summation of all the harmonic secondary structure constraints to ensure retention of well-defined secondary structure regions over the fitting process.

(3) $$U_{SS}=\sum_{\mu }k_{\mu }{\left(X_{\mu }-X_{\mu }^{0}\right)}^{2}$$

where *μ* designates the restrained internal coordinate including all the bond distances, angles, dihedral angles that relate to the well-defined secondary structure regions. *k*_*μ*_ is the force constant chosen to be applied to a particular bond, angle or dihedral angle. *X*_*μ*_ and *X*_*μ*_^0^ are the instantaneous and initial value of the restrained coordinate, respectively.

A fitting procedure is typically conducted in a multi-step manner by firstly using rigid-body fitting to optimally overlay the structure with the map followed by stages of flexible fitting wherein the magnitude of *ξ* keeps increasing from one to the next (typically varying from 0.1 kcal/mol to 0.3 kcal/mol), until the fitting has been converged as evaluated by the root mean square deviation (RMSD) and/or the cross-correlation coefficient between the simulated map generated from the fitted atomic structure and the experimental map, which is defined as:

(4) $$\rho_{SE}=\frac{\langle \left(S-\langle S\rangle \right)\left(E-\langle E\rangle \right)\rangle }{{{}_{\sigma_{S}}}_{\sigma_{E}}}$$

where *S* and *E* stand for the one particular voxel value in the simulated and experimental maps, respectively; 〈*S*〉 and 〈*E*〉 are the corresponding average voxel values; *σ*_*S*_ and *σ*_*E*_ are the corresponding deviations [47]. Trabuco et al. have provided a useful introduction to MDFF [29]. Additionally, the practical aspects of MDFF have been thoroughly explained on the developer's website (http://www.ks.uiuc.edu/Training/Tutorials/science/mdff/tutorial_mdff-html).

### 2.2 Traditional Model Building and Refinement Applied to EM

The *PHENIX* software suite [48,49] is suitable for refining experimental crystallographic data with a wide range of upper resolution bound [50], therefore, a suboptimal starting atomic model can in principle be refined into a cryo-EM map in a pseudo-crystallographic manner using *phenix.refine*. In a typical application using an EM map, the density can be segmented and fitted in artificial crystal lattices to calculate the observed structure factors, *F*_*obs*_ [50], whose amplitudes and/or phases are then used as pseudo-diffraction data for the subsequent refinement. During the refinement stage, one could opt to use a ‘black box’-like default strategy or customize the control parameters (more than 500 available), including atomic and non-atomic ones. The atomic parameters are atomic coordinates, atomic displacement parameters (ADPs), atomic occupancies and anomalous scattering terms; the non-atomic ones are used to describe bulk solvent. The parameters are combined in the expression of the total structure factor of the computational model, F_model_. In turn, the refinement is essentially a multi-step minimization of a target function that quantifies the fitness of the F_model_ to the experimental observations (*F*_*obs*_). In case of refinement against low-resolution density maps as is typical for cryo-EM maps in the 3.5–4.5 Å resolution range, the model will have to be considerably restrained (either by applying secondary structure restraints or by providing a high-resolution crystal structure as a reference model, if available). Furthermore the relative weight of the restraints versus the experimental data can be varied to reduce the risk of over-fitting and assure the overall correctness of the model.

Recently, a new xMDFF method for structural determination from low-resolution crystallographic data was introduced, which integrates the functionalities of the original MDFF method and the *PHENIX* crystallographic refinement package [51]; The MDFF protocol was modified to work with model-phased densities, wherein experimental X-ray scattering amplitudes are augmented with phases computed from an approximate initial model to produce a density map. The starting model is then flexibly fitted into the density using MDFF and this new fitted structure used to update the phases and regenerate the density map. This process is continued iteratively until convergence. In this way, xMDFF can refine initial structural models (e.g. homology models) that are quite distant from the refined structure and must undergo large-scale deformations to reach convergence. As an example of a recent application of xMDFF we point to the work of Li et al. who used the method to determine the structures *Ciona intestinalis* Voltage-sensing domain (Ci-VSD) in its active and resting forms [52].

While the successful application of *phenix.refine* using high resolution density maps include modeling the structure of *Salmonella* bacteriophage ε15 [53] and the core of hepatitis B virus [54] has been demonstrated, failing to converge on accurate atomic models often happens when the density maps are of resolution worse than ~ 3.5 Å [49]. This problem is also associated with other X-ray crystallographic tools [55–57]. To solve this problem, Dimaio et al. integrated the crystallographic refinement [50] in *phenix.refine* with Rosetta sampling to develop a hybrid refinement scheme, Rosetta–Phenix [58], which generated models with improved geometry and lower R factors [59] compared to other crystallographic refinement tools such as Phenix [48], DEN [57], and REFMAC5 [60]. More recently, a variation of this approach, tailored to refine models against high-resolution cryo-EM density maps was developed by the same group, as described below.

### 2.3 Rosetta Refinement Protocol

To address the challenges of flexible fitting into medium to near-atomic resolution cryo-EM maps Dimaio et al. developed a general Rosetta refinement protocol for generating pseudoatomic structural models [61]. This refinement protocol comprises two major stages, the first being an iterative density-guided local structural element optimization using Monte Carlo sampling, and the second an alternating between Rosetta all-atom refinement and real-space *B*-factor fitting until correlation between the map and the model converges. In the first stage, segments in the starting-model that fit poorly to the density are identified and superimposed on the endpoints by the backbone fragments from the Protein Data Bank. Variations of these fragments are obtained using Monte Carlo sampling followed by a preminimization to fit them into the density with proper constraints applied, such as coordinate constraints at the endpoints of the fragments, Ramachandran and rotameric constraints. The best fitted fragments are then selected to replace the corresponding backbone segments in the previous iteration to construct a updated structural model for a global minimization using a smooth version of the Rosetta centroid level energy function [27]. In the second stage, a real-space *B*-factor refinement is conducted using quasi-Newton optimization with restraints applied to prevent the *B* values being over-fitted [50]; and the all-atom refinement cycles are carried out using the Rosetta relax protocol [62]. The model quality can be assessed by a cross-validation measurement in reciprocal space, the expected phase error (EPE), which is independent of the quality of cryo-EM map being used for the refinement [61]. In their testing cases, the Rosetta refinement protocol largely generated more accurate models than the MDFF approach, and the refined-model accuracy was shown to be independent of the starting-model quality when using cryo-EM maps of 4.5 Å or better resolution [61]. In another recent contribution by Wang et al., the Rosetta refinement protocol was extended to enable the de novo protein structure determination using high-resolution cryo-EM maps [26].

### 2.4 Integrating SAXS Profiles into Computational Modeling

SAXS is another method, which characterizes low-resolution structural features of macromolecular assemblies. Among its advantages are tolerance to various solution conditions, relatively low concentration requirement on the sample, applicability to large size molecular assemblies, and low time/cost investment [63]. The method generates a scattering intensity profile that reveals information concerning the mass, volume, and radius of gyration of the biological assembly. Although both EM and SAXS can provide macromolecular envelopes [64,65], Fourier transform of SAXS data also yields a distribution of electron pair distances P(r) [63,66]. This constitutes a critical difference with EM, in that SAXS can sensitively discriminate among computational models, even those with the same outer envelope. All interatomic distance information is retained, even from low-populated flexible conformations. Thus, it is advantageous to develop and validate atomic models by comparing directly to the P(r) distributions and not the overall SAXS envelopes. Another important distinction is that such pseudoatomic computational models are developed through dynamics simulations and feature fully flexible relaxation of the systems. To include the SAXS data in a modeling process, it is important to compute the theoretical SAXS profile of a given atomic structural model. A variety of methods have been developed to compute theoretical SAXS profiles based on different spherical averaging, treatment of the excluded volume and hydration layer [67]. The FoXS code [68,69] is one of the popular approaches to compute the a theoretical scattering profile based on the Debye formula [70]:

(5) $$I_{q}=\sum_{i=1}^{N}\sum_{j=1}^{N}f_{i}\left(q\right)f_{j}\left(q\right)\frac{\sin\left(qd_{ij}\right)}{qd_{ij}}$$

where *I*_*q*_, the scattering intensity, is a function of the momentum transfer *q* = (4 sin *θ*)/*λ*, in which 2*θ* is the scattering angle and *λ* is the wavelength of the incident X-ray beam; *N* is the number of atoms in the system; *f*(*q*) is the form factor of one particular atom, *d*_*ij*_ is the distance between atom *i* and atom *j*.

The theoretical scattering profile can then be fitted to the experimental data by minimizing the goodness-of-fit value [71], X:

(6) $$\chi =\sqrt{\frac{1}{M}\sum_{i=1}^{M}{\left(\frac{I_{\exp}\left(q_{i}\right)-cI\left(q_{i}\right)}{\sigma \left(q_{i}\right)}\right)}^{2}}$$

where *M* is the number of points in the profile, *I*_*exp*_(*q*_*i*_) and *I*(*q*_*i*_) are the experimental and theoretical profiles, respectively. *σ*(*q*_*i*_) is the experimental error, and *c* is the scaling factor. It is worth noting that the X values are comparable only for the same experimental profile, since the experimental error is different with different sets of experimental profiles.

Experimental scattering profiles can be informative for modeling in different ways. First, the profile can be used as a reference to assess models. For example, a straightforward comparison between the computed SAXS profile of a crystal structure and experimental profile can reveal possible different oligomeric states or structural features due to a difference in environments between crystal and solution [72,73]; the conformation from a number of possible homology models can be distinguished by using SAXS data [74]. Alternatively, the SAXS data can be incorporated into the modeling process by fitting a single perturbed conformation to the profile [75] or modeling an ensemble of conformations [72]. It is often preferable to fit the profile to an ensemble of conformations when the macromolecule or complex is flexible in solution [76]. Among the methods that have been developed to generate the ensemble from a pool of candidate conformations, the EOM [77] and minimal ensemble search (MES) [78] are particularly useful. Specifically, MES uses a genetic algorithm to select a small subset of weighted conformations that optimally represent the SAXS I(q) profile. Goodness-of-fit between computed and experimental SAXS profiles is measured by $X_{\mathrm{free}}$ [79], which gives a noise-reduced assessment of the fit. The criterion used to prevent over-fitting is including in the ensemble as few conformations as necessary to minimize $X_{\mathrm{free}}$. A variety of parameters such as RMSD, normalized spatial discrepancy (NSD), maximal diameter (D_MAX_), and radius of gyration (Rg) from the minimal ensemble can be used to compare with those from the original conformation pool to shed light on the flexibility of the macromolecule or complex in solution.

Other computational approaches use SAXS profiles directly in modeling and don't involve filtering of preexisting MD ensembles. For example, Förster et al. incorporated SAXS profile into Monte Carlo sampling in which new conformations are accepted or rejected based on Metropolis criterion of SAXS-based $X^{2}$ statistics [80]; Gorba et al. used linear combination of low frequency normal modes to deform the structural models in order to conform to the pair distribution function derived from experimental SAXS profile [81]. More recently, Chen et al. reported a method with which the dynamic trajectory of a protein in solution can be modeled by incorporating SAXS or SWAXS (small and wide angle X-ray scattering) information as a differentiable energetic restraint into explicit solvent MD simulation [82,83]. These methods greatly enhance the power of SAXS in determining multi-functional states of biological entities. A valuable review of SAXS-based integrative modeling methods was given by Schneidman-Duhovny et al. [67].

## 3 Example Applications

### 3.1 DNA Repair Complex of Human Rad9-Hus1-Rad1/FEN1/DNA

PCNA and Rad9–Hus1–Rad1 (9-1-1) are sliding clamps specialized in DNA replication and DNA repair, respectively. Association and handoff of DNA-editing enzymes, such as flap endonuclease 1 (FEN1), with these clamps are critical events of which the mechanistic details are poorly understood. To provide an atomistic level description of the complexes of FEN1 with its DNA substrate in the presence of either PCNA or 9-1-1 and to reveal the structural foundation of functional differences, negative stain EM and an integrative computational approach were used, in which different component crystal structures, single-particle EM data and modeling were used to obtain atomistic models of each complex. The EM data were collected on the Fei Tecnai F20 at 80,000 × magnification (1.5 Å/pixel) in low dose (20 e^−^/Å^2^) with a Gatan 4 K × 4 K pixel CCD camera (15-μm pixel size). Specifics on the software used for data collection, image processing and 3D reconstruction are detailed in the original reference [28].

**Fig. 1:**
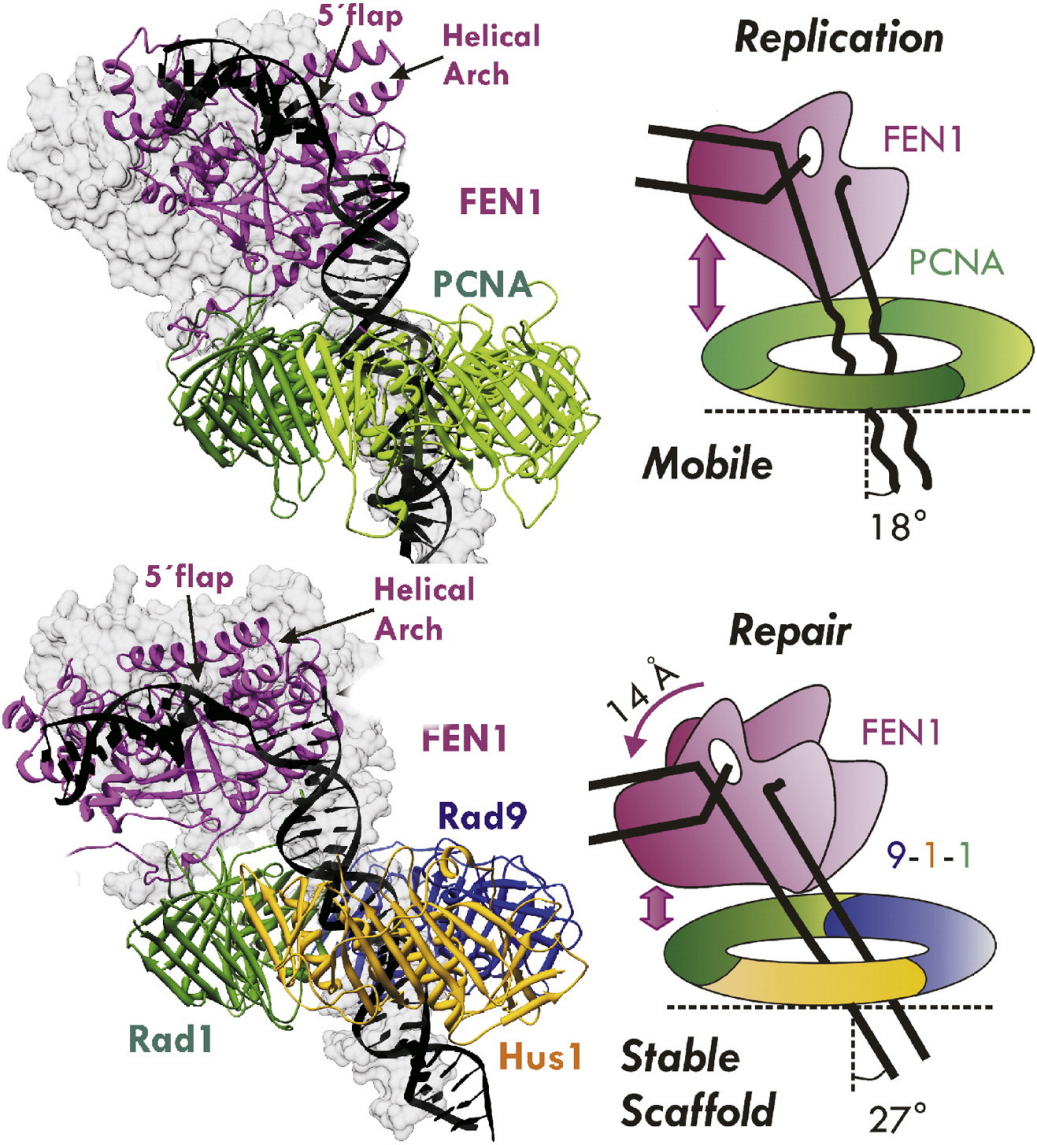
Active orientation of DNA editing enzymes revealed in models of FEN1 with sliding clamps and DNA. Computational models of FEN1 with PCNA and 9-1-1 were developed based on one of the stabilized positions of FEN1 in a DNA-free PCNA crystal structure. Modeling revealed that the sliding clamps tilted the DNA toward FEN1. The PCNA and 9Δ-1-1 complexes are shown as cartoons. PCNA is shown in green, FEN1 in purple, Rad1 in green, Hus1 in yellow, Rad 9 in blue, and DNA in black. The gray surfaces are the FEN1/DNA from the original starting models. The surfaces for the two clamps in the starting models were omitted for clarity. (For interpretation of the references to color in this figure legend, the reader is referred to the web version of this article.)

The modeling process was started by overlaying FEN1 from the FEN1/DNA structure (PDB access code 3Q8L) and PCNA/FEN1 structure (PDB access code 1UL1). A double-stranded B-form DNA (dsDNA) extension was then introduced on the 3′ flap side to pass through the PCNA ring. An initial model of 9-1-1/FEN1/DNA was then generated by replacing PCNA in PCNA/FEN1/DNA with 9-1-1. FEN1 interacts with the Rad1 subunit in this complex based on previous experimental evidence. Both initial models were then refined through ~ 120 ns MD simulation to fully relax the systems, followed by pairwise RMSD clustering analysis to select the centroid of each dominant cluster as final model, shown in Fig. 1.

**Fig. 2:**
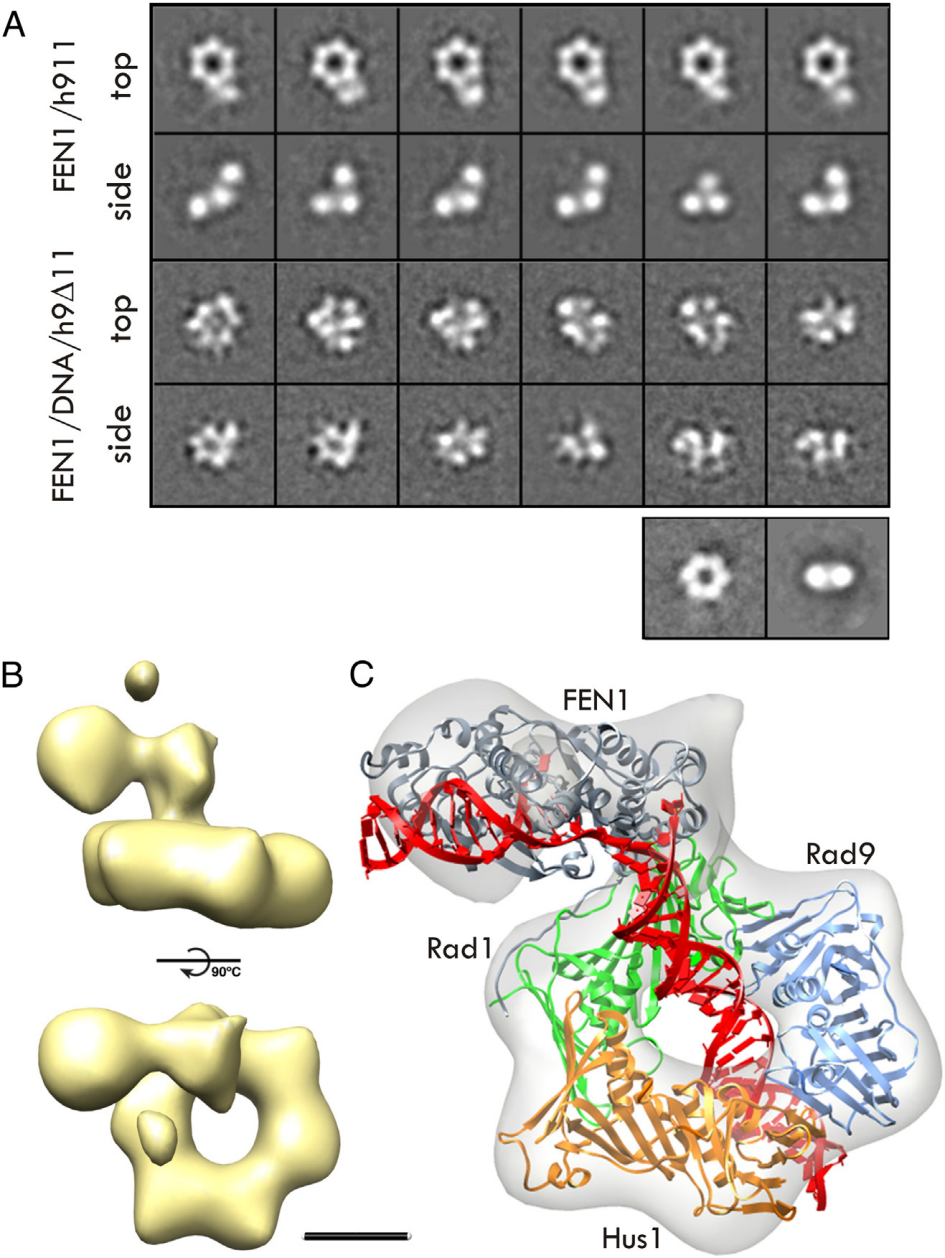
Single-particle EM analysis shows how FEN1 interacts flexibly with 9-1-1 and adopts a fixed position on 9Δ-1-1 in the presence of the DNA substrate. (A) Representative reference-free 2D class averages (top and side views) for the 9-1-1/FEN1 binary complex are compared with those corresponding to the 9Δ-1-1/FEN1/DNA ternary complex. Top and side views of the 9-1-1 complex are shown. (B) Side and top views of the 9Δ-1-1/FEN1/DNA 3D reconstruction. (C) MDFF flexible fitting of the 9Δ-1-1/FEN1/DNA complex into the 3D map of the ternary complex.

Using single-particle EM, the structural features of the binary complexes of 9-1-1/FEN1 and the ternary complex of 9-1-1/FEN1/DNA were revealed by the reference-free 2D class averages as shown in Fig. 2A. The computational model of 9-1-1/FEN1/DNA was then filtered at 20 Å to assign relative orientations to the different experimental views of the assembly. A final 3D reconstruction of the 9-1-1/FEN1/DNA complex at a resolution of 18 Å was then obtained using 3D refinement with iterative projection matching [84,85] (Fig. 2B). The atomistic model was then flexibly fitted into the EM map using MDFF. Due to the difficulty in visualizing DNA density with negative staining, the DNA was not included in the MDFF process. In the end, the fitted atomic model of 9-1-1/FEN1/DNA left fewer than 300 atoms outside of the EM map (at a threshold of 3.6), showing an excellent agreement with the map (Fig. 2C). It is worth noting that the EM map supports the observation that FEN1 is tilted toward the Rad1 subunit in the computational model.

**Fig. 3:**
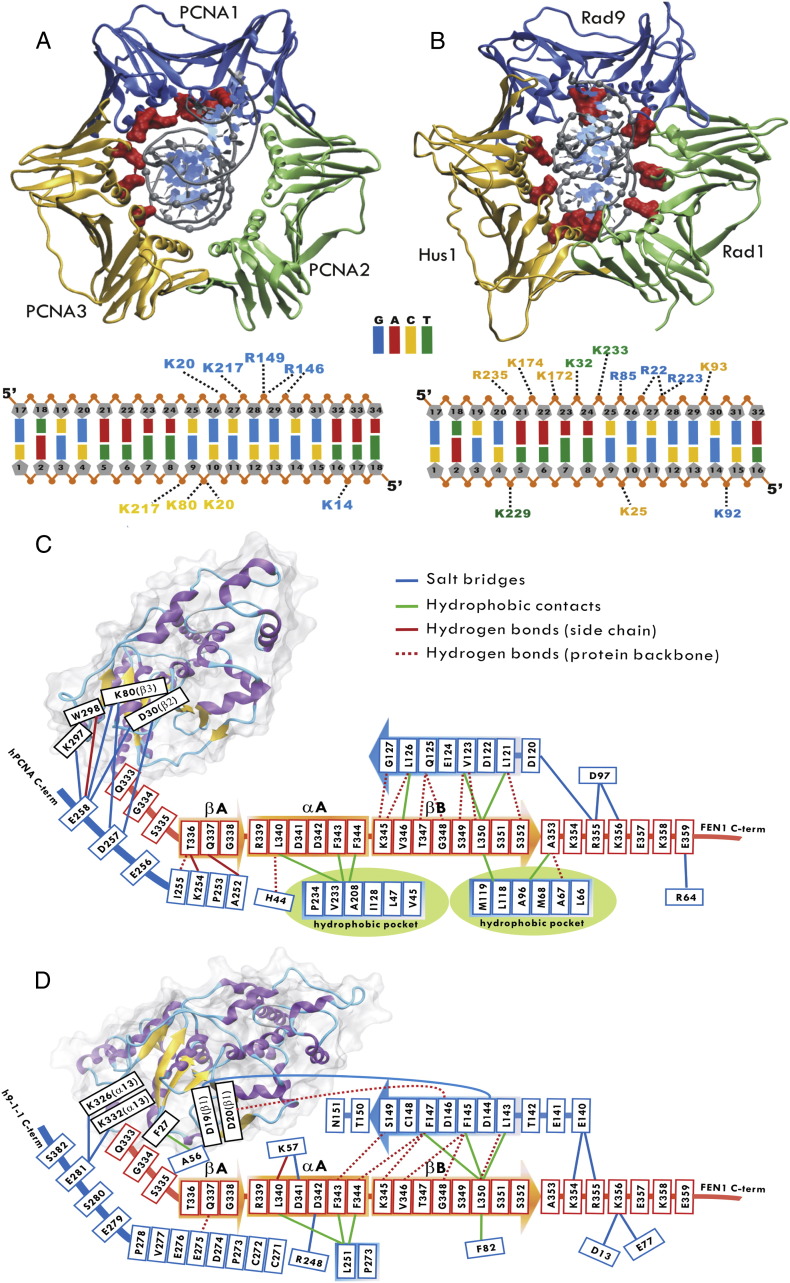
Two distinct binding modes of the PCNA/FEN1/DNA and 9∆-1-1/FEN1/DNA complex. A–B) Cartoon representations of PCNA and 9-1-1 binding to dsDNA, colored in blue for Rad9 and PCNA1, yellow for Hus1 and PCNA3 and green for Rad1 and PCNA2. The dsDNA phosphodiester groups and basic residues on the inner surface of PCNA and 9-1-1 are shown in gray spheres and red surfaces, respectively. Schematic representations of C) PCNA/FEN1 and D) 9∆-1-1(Rad1)/FEN1 interfaces and contacts. Secondary structure elements are shown for the FEN1 C-terminal tail in orange and sliding clamp (PCNA/Rad1) in blue. Ribbon representations of the core of FEN1 with secondary structure elements are labeled. Hydrophobic pockets on the PCNA surface are indicated in green. (For interpretation of the references to color in this figure legend, the reader is referred to the web version of this article.)

Detailed analysis of the contacts of clamp/DNA or clamp/FEN1 with the models has illuminated the structural basis for their functional specialties (Fig. 3). FEN1 adopts an overall upright position on the clamp's surface, with its DNA substrate passing through the ring at a tilted angle; in either case, the upstream DNA passes through the 9-1-1 ring at an even greater angle than it does through the PCNA ring. The DNA also forms more persistent contacts with the inner layer of clamp in 9-1-1/FEN1/DNA. The distinct DNA interactions with these clamp proteins are consistent with the functional difference between the two complexes: PCNA needs to be mobile on DNA in conjunction with replicative polymerases, while 9-1-1 serves as a temporary scaffold for DNA repair at specific sites. Interesting differences in the interactions of clamp/FEN1 for each complex were also observed beyond the conservative, inter-domain connector loop – PCNA-interacting protein motif (PIP) interaction, often referred to as “IDCL-PIP box interaction”. The PCNA/FEN1 interface features two stable hydrophobic pockets in the C-terminus of PCNA, which interact with the PIP box in the C-terminus of FEN1 (Fig. 3C). In contrast, the Rad1/FEN1 interface lacks the corresponding hydrophobic interactions (Fig. 3D). This difference rationalizes a previous report that the exact C-terminal residues responsible for stimulation of FEN1 by the two clamps are distinct [86].

### 3.2 Modeling Ubiquitin-modified PCNA Using SAXS Data

Post-translational modification of PCNA by ubiquitin is essential for PCNA to recruit the specialized polymerase needed to carry out translesion DNA synthesis (TLS) – a major mechanism to bypass DNA damage sites, which stall replication by classical DNA polymerase. The mono-ubiquitylated PCNA (PCNA-Ub) governs the step of recruiting TLS polymerase and the conformational switch between the replicative and translesion polymerase. It does so by providing additional binding surfaces for interaction with their ubiquitin-biding motifs [87–89]. X-ray crystallography studies of PCNA mono-ubiquitylated at Lys164 (PCNA_K164_-Ub) have revealed that the ubiquitin interacts with PCNA on the back face of PCNA (as opposed to the front face of PCNA, where most PCNA-interacting proteins bind) [90]. Crystallography had also revealed that paradoxically, the ubiquitin surface engaged in PCNA interactions was the same as the surface implicated in translesion polymerase binding. Apparently, a dynamic process of exposing this binding surface of ubiquitin is necessary for the recruitment and switching to the TLS polymerase. This finding implied a degree of flexibility inherent in the complex. To address this segmental flexibility, we combined multi-scale computational modeling and SAXS to reveal alternative positions for ubiquitin on PCNA, distinct from the crystal structure [30].

**Fig. 4:**
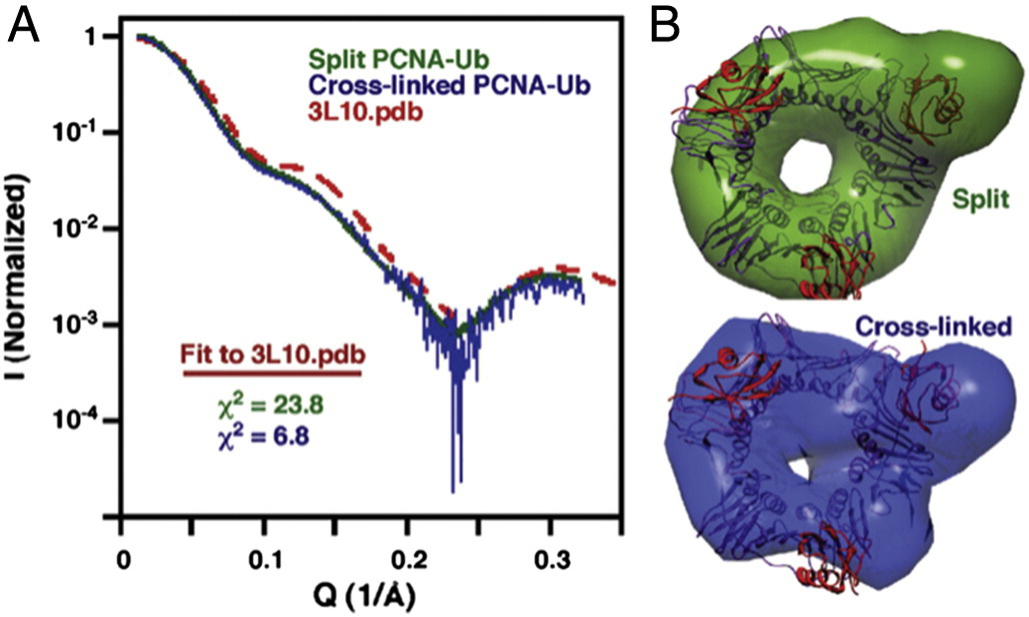
SAXS analysis of PCNA_K164_-Ub in solution suggests that ubiquitin is not exclusively oriented in the position determined by crystallography. (A) SAXS curves. (B) Molecular envelope derived from SAXS data analysis of split-fusion or cross-linked PCNA_K164_-Ub.

Yeast PCNA_K164_-Ub was obtained using either split-fusion construct [90] or chemical cross-linking with mutant PCNA (K164C). SAXS data of both constructs in solution were compared, showing nearly identical profiles (Fig. 4A). Ab initio 3D shapes of both constructs were generated, indicating the core, torroidal structure of PCNA, and also the protruding part comprising the ubiquitin moiety. This conjunction of ring-plus-protrusion do not agree well with the position of ubiquitin in the crystal structure (PDB accession code 3L10) (Fig. 4B). However, fitting the solution SAXS profiles to the crystal structure profile generated a high value of χ (Fig. 4A), which in combination with the observation of discrepancies between the ab initio 3D shape and crystal structure favors previously unrevealed conformational states of ubiquitin in the PCNA_K164_-Ub complex in solution.

**Fig. 5:**
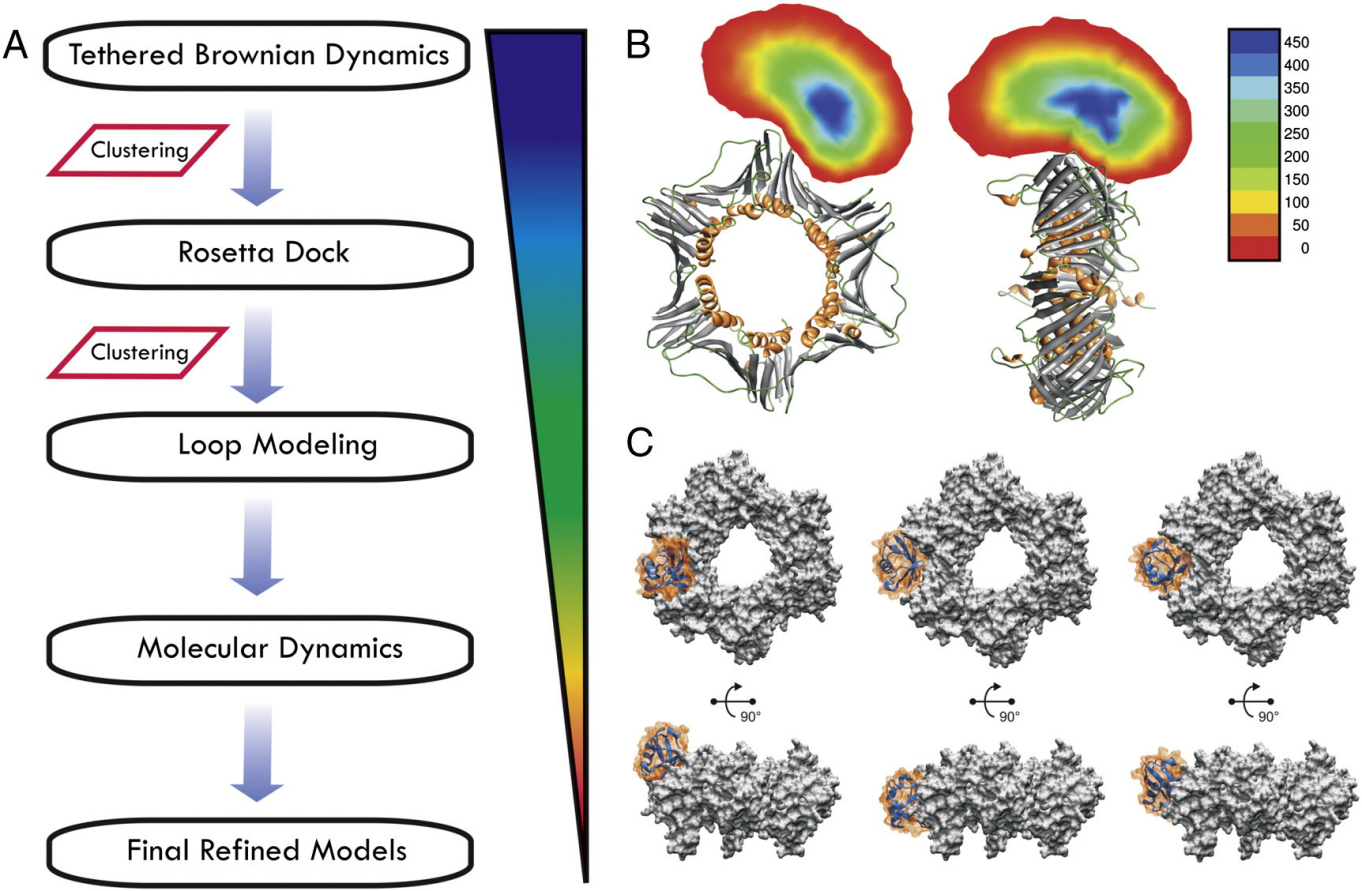
Flowchart of the modeling protocol for PCNA_K164_-Ub. (A) The strategy to generate models for PCNA with covalently bound ubiquitin. (B) The positions of the covalently bound Ub heavy atoms in 6837 frames from a 34-μs TBD simulation were binned and displayed relative to PCNA; the number of frames in each bin is color coded as from smallest (red) to largest (blue). (C) PCNA_K164_-Ub complex identified from multi-scale refinement in surface representation.

To examine the conformational space of Ub on PCNA more systematically, a successive, computational modeling approach was adopted by combining tethered Brownian dynamics (TBD) [91], protein–protein docking (using RosettaDock [92–94]), flexible loop modeling (using ModLoop [95,96]), and MD simulation (Fig. 5). First, an extensive TBD simulation of 34 μs identified a bound state of PCNA-Ub, based on electrostatic and shape complementarity. The resulting conformational ensemble was then clustered into 90 clusters in order to select the centroids for local protein–protein docking; in this process side-chain packing was allowed, as opposed to side-chain rigidity imposed in TBD. The distinct, dominant docking modes from the top three Rosetta-scoring models showed Ub situated in the large cleft defined by a β-sheet that forms the subunit–subunit interface of PCNA. As the tethering peptide in both TBD and RosettaDock calculations was included implicitly, the models were then completed by including the linker (Ub residues 72–76) into the structures obtained from clustering with RosettaDock (Fig. 5) using Modloop, and subsequently refined through ~ 25 ns all-atom, explicit solvent MD simulations.

**Fig. 6:**
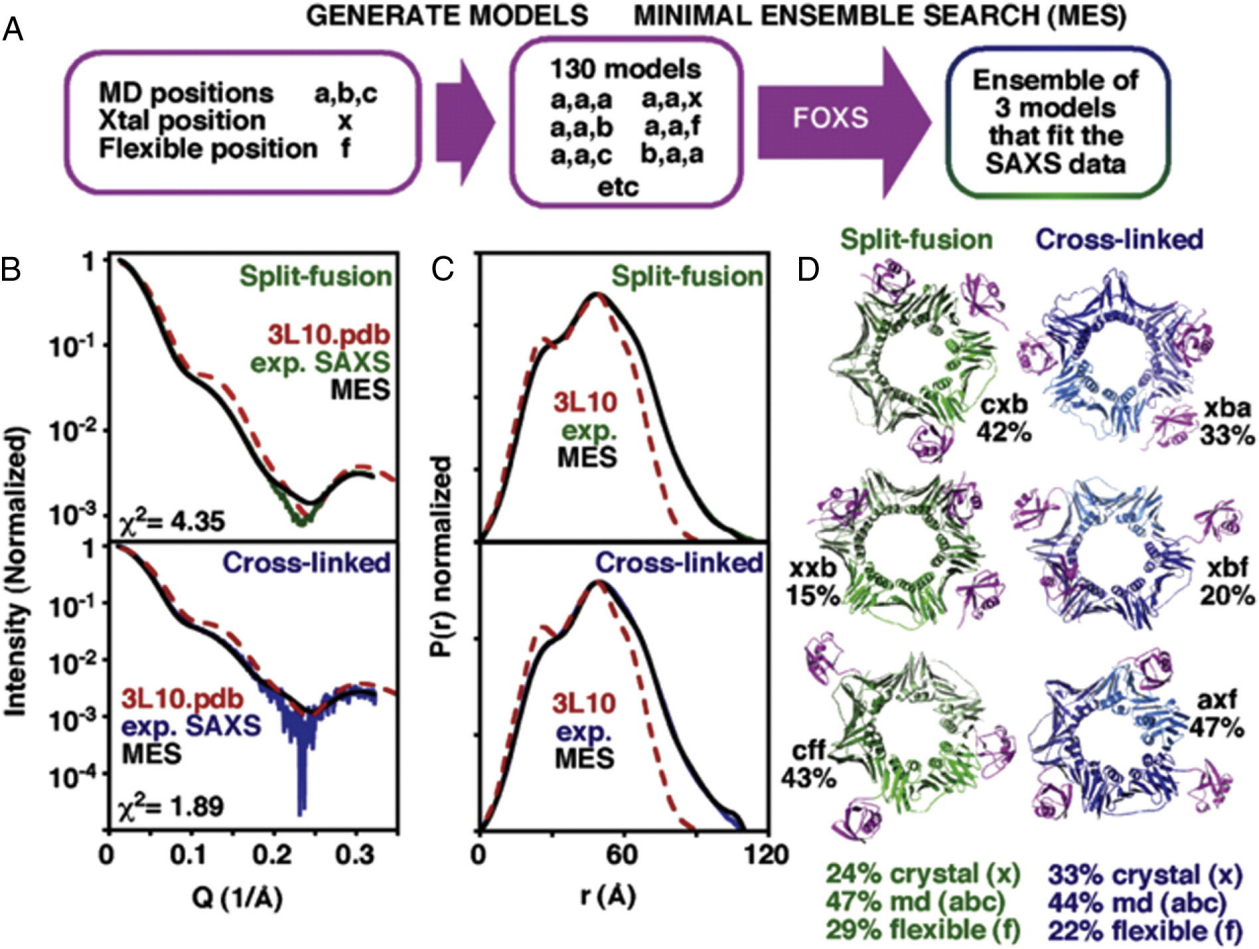
A MES ensemble of discrete Ub positions on PCNA best fit the experimental SAXS data for split-fusion (green) and cross-linked (blue) PCNA_K164_-Ub. (A) Schematic showing the MES methodology. (B) The scattering curve of the best MES ensemble fits the experimental scattering data better than the crystal structure 3L10.pdb. (C) P(r) plots. (D) Structures of the three models that as an ensemble best fit the experimental scattering curve are shown. (For interpretation of the references to color in this figure legend, the reader is referred to the web version of this article.)

Flexible positions of Ub on PCNA were then identified using BILBOMD [78] with the models from the MD simulation. This flexible position of Ub, along with the original MD positions, and the position observed in the crystal structure were then permuted on the homotrimer of PCNA to generate 130 PCNA_K164_-Ub PDB models, where each PCNA is modified by three Ubs. MES was then applied using this pool of models to fit either the split-fusion or the cross-linked PCNA_K164_-Ub SAXS profile. An ensemble of three models for each of the experimental constructs was identified, with the Ub being 25–30% in the crystallographic position, 40–50% in the computationally determined positions, and 25–30% flexible positions (Fig. 6). The result suggests a segmental flexibility of the Ub in PCNA_K164_-Ub, meaning that in solution Ub can adopt a number of discrete interchangeable positions on the surface of PCNA. This segmental flexibility of the Ub moiety on PCNA-Ub provides a variety of distinct positions capable of forming complexes with TLS polymerase, and, accordingly spatially organizes the PCNA-Ub interacting proteins for either efficient DNA replication or repair. These novel positions provided a rationalization for perplexing biochemical data e.g. explained the effects of mutations originally identified in genetic screens and known to interfere with TLS. The computationally derived positions, in an ensemble with the crystallographic position, provided the best fit to the solution scattering. The finding of new docking sites and the positional equilibrium of PCNA-Ub occurring in solution provided unexpected insight into the question of how Ub may help transition the TLS Pol from the back to the front side of PCNA to exchange with the replicative Pol [30].

**Fig. 7:**
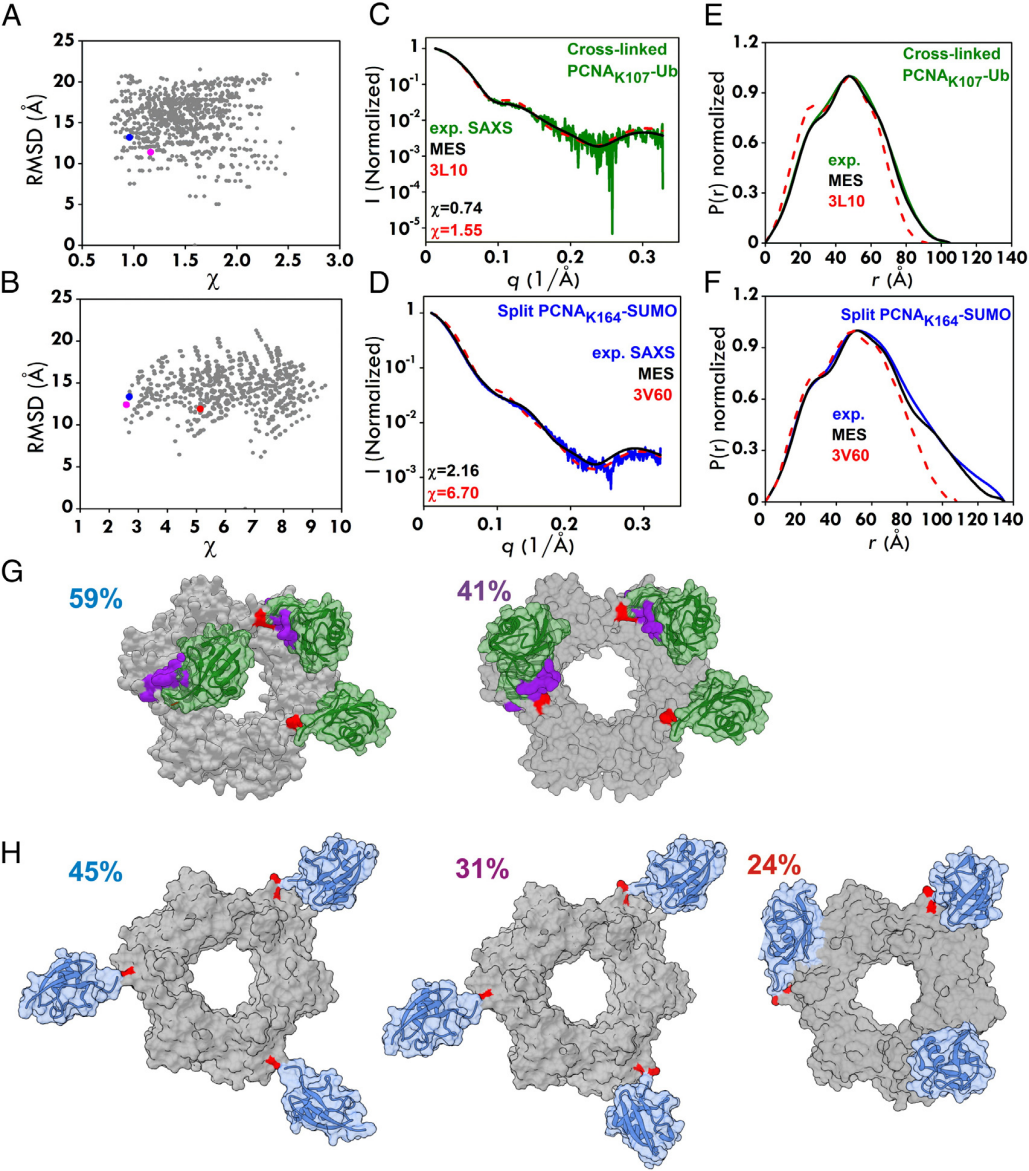
Ub primarily adopts docked positions in PCNA_K107_-Ub while SUMO occupies extended positions in PCNA_K164_-SUMO (A,B) χ values for the triplet PCNA_K107_-Ub and PCNA_K164_-SUMO structures plotted against RMSD. Conformations selected by MES are highlighted in blue, magenta and red, respectively. (C,D) Overlaid SAXS profiles. (E,F) Overlaid P(r) plots. (G,H) The most populated atomic structures from MES analysis of PCNA_K107_-Ub and PCNA_K164_-SUMO in surface representation. The K107 and K164 attachment points are depicted in red. PCNA, Ub and SUMO are shown in gray, green and blue, respectively. (For interpretation of the references to color in this figure legend, the reader is referred to the web version of this article.)

We have recently extended this work to provide a common hybrid modeling/SAXS framework and examined K107-Ub and SUMOylated PCNA [84]. The biological functions of the small ubiquitin-related modifier SUMO appear to be even more diverse, ranging from nuclear transport to signal transduction, transcription, and genome stability [85]. Sumoylation of PCNA occurs on two lysines, predominantly on K164 and to a lesser extent on K127. Attachment of SUMO can induce a variety of cellular outcomes but often its mode of action remains poorly understood. To explore the overall architecture and flexibility of yeast PCNA_K107_-Ub and PCNA_K164_-SUMO complexes, we examined solution conformations with small-angle X-ray scattering (SAXS). Yeast PCNA_K107_-Ub was produced using chemical cross-linking with a K107C mutant PCNA. The PCNA_K164_-SUMO complex was formed by split-fusion [76]. Experimental SAXS curves (Fig. 7), along with a compaction observed in the pair distribution P(r) plot, show the three complexes adopt conformations with different levels of compactness in solution. The χ fit of the SAXS model to existing crystal structures (with PDB codes 3L10 and 3V60) produced high values, consistent with significant discrepancies between the observed structures in solution and in the crystalline environment.

To further probe the conformational differences of the PCNA_K107_-Ub and PCNA_K164_-SUMO complex implied by SAXS experimental data, we created models using a recently developed protein conjugated docking module in Rosetta 3.4. The protocol involved searching the conformational space available to ubiquitin or SUMO when chemically conjugated via an isopeptide bond to PCNA. Sampling proceeded with the standard Rosetta Metropolis-Monte Carlo search protocol [86,87]. For the isopeptide linker, torsions sampled included the χ angles of Lys107 or Lys164 of PCNA, the isopeptide bond and both Φ and Ψ angles for the Gly76, Gly75 and Arg74 of ubiquitin (Gly98, Gly97 and Ile96 of SUMO). The lowest-scoring structurally distinct models from the Rosetta output were selected and refined using all-atom explicit solvent molecular dynamics (MD). The conformations easily departing from the PCNA surface were excluded during MD refinement. Twelve positions for PCNA_K107_-Ub (including 3 detached flexible Ub positions identified by averaging from the MD ensemble) and twelve positions for PCNA_K164_-SUMO (including the 3V60 X-ray structure and 3 detached flexible SUMO positions) were used to generate models with three ubiquitin or three SUMO moieties linked to homotrimeric PCNA. The trimeric models were then used for comparison to the experimental SAXS data. Theoretical SAXS profiles for all triplet models of the modified complex were computed with the program FOXS and fitted to the experimental profiles. Fig. 7A, B shows computed χ values for PCNA_K107_-Ub and PCNA_K164_-SUMO as a function of Cα RMSD for each conformation. A MES [64] was then utilized to identify a small subset of conformations that as an ensemble best fits the scattering data. The fit to the experimental data improved significantly (Fig. 7C,D). This study demonstrated that Ub adopts discrete docked binding positions on PCNA and the position of ubiquitin attachment, 107 versus 164, alters conformation. In contrast to Ub, SUMO adopts extended flexible conformations on PCNA by simple tethering (Fig. 7G,H). The distinct structural features can be explained by the opposite surface electrostatic potentials of SUMO and Ub, and present different accessibility of interacting surface for partner proteins of Ub-PCNA and SUMO-PCNA. This observation elucidates the structural basis for the different functional involvement of Ub-PCNA and SUMO-PCNA in DNA repair pathway regulation.

## 4 Summary and Outlook

Studies in structural biology have substantially enhanced our understanding on the molecular mechanisms of many biological pathways by way of solving structures at different resolution. While X-ray and NMR are capable of generating structures at atomic resolution, they are limited in their ability to access large flexible biomolecular assemblies. Cryo-EM and SAXS, on the other hand, are well suited to generating structural data from low- to medium to near-atomic resolution, without substantial limitation on the size of the molecule/assembly. Integrative modeling takes advantage of the available experimental data at different levels of resolution, and combines them in complementary ways, which enable retention of the highest resolutions while yielding an accurate “overall picture”.

Experimental data from other studies, which reveal interaction information, can also find their way into the final model. Models resulting from integrative modeling often enhance our understanding of the function of the molecule/assembly from a mechanistic point of view, as illustrated by the modeling applications presented in this review. The applicability and power of integrative modeling approaches to DNA replication machinery are demonstrated further by recent studies of the complexes of single-stranded DNA with replication protein A (RPA) [97,98] utilizing both SAXS and NMR data. Thus, integrative modeling is an emerging area with great promise as evidenced by the sheer variety of methods, ever-expanding modeling codes e.g. the integrative modeling platform (IMP) [23], the the inferential structure determination (ISD) framework [99], HADDOCK [100] and RNABuilder [101], and exemplary applications reported. Regardless of what sources of experimental constraints a particular method or software framework is able to incorporate, if it provides insightful models it is of value. Integrative modeling is a composite method, not an ultimate goal, a sort of in silico microscope enabling us to discern atomic-level mechanisms underlying biological functions.
